# Effect of amorphous silica nanoparticles on *in vitro *RANKL-induced osteoclast differentiation in murine macrophages

**DOI:** 10.1186/1556-276X-6-464

**Published:** 2011-07-22

**Authors:** Hiromi Nabeshi, Tomoaki Yoshikawa, Takanori Akase, Tokuyuki Yoshida, Saeko Tochigi, Toshiro Hirai, Miyuki Uji, Ko-ichi Ichihashi, Takuya Yamashita, Kazuma Higashisaka, Yuki Morishita, Kazuya Nagano, Yasuhiro Abe, Haruhiko Kamada, Shin-ichi Tsunoda, Norio Itoh, Yasuo Yoshioka, Yasuo Tsutsumi

**Affiliations:** 1 Laboratory of Toxicology and Safety Science, Graduate School of Pharmaceutical Sciences, Osaka University, 1-6 Yamadaoka, Suita, Osaka 565-0871, Japan; 2 Laboratory of Biopharmaceutical Research (Pharmaceutical Proteomics), National Institute of Biomedical Innovation, 7-6-8 Saito-Asagi, Ibaraki, Osaka 567-0085, Japan; 3 The Center for Advanced Medical Engineering and Informatics, Osaka University, 1-6 Yamadaoka, Suita, Osaka 565-0871, Japan; 4 Laboratory of Biomedical Innovation, Graduate School of Pharmaceutical Sciences, Osaka University, 7-6-8 Saito-Asagi, Ibaraki, Osaka, 567-0085, Japan

**Keywords:** silicon dioxide, nanoparticle, osteoclast differentiation

## Abstract

Amorphous silica nanoparticles (nSP) have been used as a polishing agent and/or as a remineralization promoter for teeth in the oral care field. The present study investigates the effects of nSP on osteoclast differentiation and the relationship between particle size and these effects. Our results revealed that nSP exerted higher cytotoxicity in macrophage cells compared with submicron-sized silica particles. However, tartrate-resistant acid phosphatase (TRAP) activity and the number of osteoclast cells (TRAP-positive multinucleated cells) were not changed by nSP treatment in the presence of receptor activator of nuclear factor κB ligand (RANKL) at doses that did not induce cytotoxicity by silica particles. These results indicated that nSP did not cause differentiation of osteoclasts. Collectively, the results suggested that nanosilica exerts no effect on RANKL-induced osteoclast differentiation of RAW264.7 cells, although a detailed mechanistic examination of the nSP70-mediated cytotoxic effect is needed.

## Introduction

Recently, amorphous silica nanoparticles (nSP) with a controlled particle size below 100 nm have been a focus of investigations in various fields of industry. nSP have been used in a number of different industrial applications such as medicine, cosmetics and foods. In addition, the usability of nSP has been demonstrated in the oral care field, e.g. as a polishing agent and/or as a remineralization promoter for teeth [1,2]. It has been reported that about 20% of toothpastes contain nSP. Because nSP have already become commonly used materials, it is difficult to imagine our daily life without them. Furthermore, given the recent use of smaller-sized and/or well-dispersed silica particles in various fields, it is expected that the use of these particles will increase in the future. On the other hand, there have been many reports that nSP exert biological effects that are not induced by conventional silica particles [3,4], although the reasons for the effects of particle size on biological responses were unclear. There are growing concerns about the safety of nSP [5]. However, current risk analyses do not yet focus sufficiently on the particle sizes. Accordingly, there is a compelling need to clarify the biological and cellular responses induced by different particle sizes. To ensure the safe production and use of nSP, it is very important to collect safety information on them via properly designed studies, taking into consideration exposure levels and cellular responses.

Our group carried out a previous study of the safety of nSP and revealed that surface unmodified nSP could pass though the skin barrier, migrate into the bloodstream and circulate throughout the entire body [6]. This suggests that nSP may be absorbed through the oral mucosa easily when nSP, including oral care products, are used. Furthermore, it is possible that nSP circulating in the blood can reach the alveolar bone, which is presented in the submucosal layer, as well as various cells such as macrophages and osteoblasts. In particular, macrophages are known as multifunctional cells, as they can function not only as immunocompetent cells, but also as pre-osteoclasts [7]. Osteoclasts that resorb bone play an important role in bone remodelling. Osteoclastogenesis involves complex pathways with intricate relationships between multiple signalling molecules. In particular, receptor activator of nuclear factor κB ligand (RANKL) is known to be a key molecule that initiates osteoclast formation [8]. In addition, reactive oxygen species (ROS) and pro-inflammatory cytokines such as interleukin (IL)-1, IL-6, IL-8 and tumour necrosis factor alpha are potent stimulators of osteoclast formation and activity [9-11]. Our studies revealed that nSP induced high ROS and pro-inflammatory cytokine production, and these cellular responses may induce excess osteoclast differentiation. Acceleration of osteoclast differentiation, that is excess bone resorption, accelerates the onset of osteoporosis, arthritis and periodontal disease [12-14]. Therefore, we consider that it is necessary to estimate nSP-induced effects on osteoclast differentiation. Furthermore, because we indicated previously that nSP induced different cellular responses from submicron-sized silica particles, such as those described above, it was of interest to analyse the effects of particle size on osteoclast differentiation. Here, we investigate nSP-induced effects on osteoclast differentiation and the relationship between particle size and these effects.

## Experimental procedures

### Silica particles

Suspensions of amorphous silica particles (Micromod Partikeltechnologie GmbH, Warnemuende, Germany) (25 and 50 mg/ml) were used in this study; particle size diameters were 70, 300 and 1,000 nm (designated as nSP70, nSP300 and mSP1000, respectively). Silica particle suspensions were stored at room temperature. The suspensions were sonicated for 5 min and then vortexed for 1 min immediately prior to use.

### Cell culture

The mouse macrophage cell line RAW264.7 was obtained from the American Type Culture Collection (ATCC, Manassas, VA, USA). RAW264.7 cells were cultured in Dulbecco's Modified Eagle Medium supplemented with 10% heat-inactivated fetal calf serum (FCS), 1% antibiotic-antimycotic mix stock solution (Invitrogen Corporation, Carlsbad, CA, USA). All cultures were incubated at 37°C in a humidified atmosphere with 5% CO_2_.

### Cytotoxicity test

The cytotoxicity of silica particle-treated RAW264.7 cells and untreated cells was assessed using a WST-8 assay. Cells, 1.5 × 10^3^, were cultured with varying concentrations of silica particles diluted with medium for 5 days at 37°C and 10 μl of Cell Count Reagent SF (Nacalai Tesque, Inc., Kyoto, Japan) was then added into each well. After 1 h, absorbance was measured at 450 nm (reference, 650 nm) using a microplate reader (Mithras LB940; BERTHOLD TECHNOLOGIES GmbH & Co. KG, Bald Wildbad, Germany).

### TRAP staining and activity assay

Osteoclast differentiation of silica particle-treated RAW264.7 cells was assessed by tartrate-resistant acid phosphatase (TRAP) staining and activity. RAW264.7 cells were suspended in phenol red-free α-MEM containing 10% heat-inactivated FCS, 1% antibiotic-antimycotic mix stock solution. Cells numbering 2 × 10^4 ^per well were cultured in 48-well plates with 10 μg/ml of silica particles and RANKL (30 ng/ml) diluted with phenol red-free medium for 5 days. TRAP staining was carried out using a TRAP/alkaline phosphatase (ALP) stain kit (Wako Pure Chemicals Industries Ltd., Osaka, Japan) according to the protocol provided in the kit. Images of TRAP-positive multinucleated cells (number of nuclei > 3) were captured with a microscope. To measure TRAP activity, cells were fixed with 10% formalin for 10 min and 95% ethanol for 1 min, and then 100 μl of citrate buffer (50 mM, pH 4.6) containing 10 mM sodium tartrate and 5 mM p-nitrophenylphosphate (Sigma-Aldrich Corporation, St. Louis, MO, USA) was added to the wells containing fixed cells in the 48-well plates. After incubation for 1 h, enzyme reaction mixtures in the wells were transferred to new plates containing an equal volume of 0.1 N NaOH. Absorbance was measured at 410 nm using a microplate reader (Mithras LB940; BERTHOLD TECHNOLOGIES GmbH & Co. KG, Bald Wildbad, Germany). Each experiment was performed in triplicate.

### Statistical analysis

All data are reported as the mean ± SD. The significance of variation among different groups was determined by one-way ANOVA. Differences between the experimental and control groups were determined using the Bonferroni test. *P *< 0.05 was considered significant.

## Results and discussion

This study evaluated the effects of amorphous silica particles on osteoclast differentiation of macrophage cells, using silica particles of 70 (nSP70), 300 (nSP300) and 1,000 nm (mSP1000) in diameter. We previously confirmed that these silica particles were spherical, and the primary particle sizes were approximately uniform [6]. In addition, none of the silica particles used in this study were modified with any functional groups, and their surfaces were nonporous. The mean particle size in PBS suggested that the silica particles used in this study remained as stable well-dispersed particles in solution, i.e. they did not aggregate [6].

**Figure 1:**
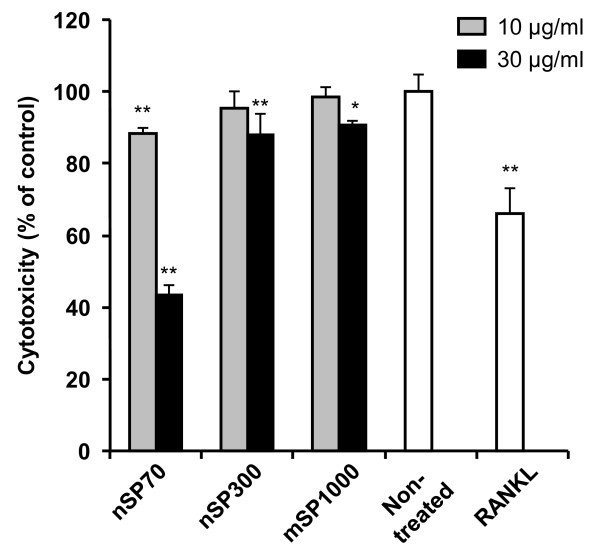
**Effect of silica particles on cytotoxicity**. The cytotoxicity of RAW264.7 cells after incubation with nSP70, nSP300 or mSP1000 for 5 days was evaluated using the WST-8 assay. The percentage increase in cytotoxicity was calculated relative to the negative control. Data are expressed as the mean ± SD (*n *= 3). *P < 0.05, **P < 0.01 vs non-treated.

To evaluate the cytotoxicity induced by silica particle treatment of the mouse macrophage cell line RAW264.7 cells, a WST-8 cell proliferation assay was carried out. The results showed that 30 μg/ml of nSP300 and mSP1000 treatment for 5 days did not induce cytotoxicity in RAW264.7 cells. In contrast, nSP70 treatment induced higher cytotoxicity (about 40% viability of non-treated cells) at 30 μg/ml, although 10 μg/ml of nSP70 treatment produced only marginal cytotoxicity (Figure 1). These results indicated that decreasing the silica particle size to below 100 nm increased the cytotoxicity significantly. We have confirmed that the number of silica particles ingested by cells increases as the particle size decreases, and only nSP70 invaded into the nuclei of dendritic cells which, along with macrophages, have a phagocytic capacity [15]. We also found that only nSP70 invaded into the nucleus, in other words the intracellular localization of nSP70 differed from that of nSP300 and mSP1000 [15]. From these results, it was suspected that differences in the number of ingested silica particles and/or in their intracellular localization were significant factors in their observed cytotoxicity in RAW264.7 cells. Results of this experiment confirmed that not all silica particles induced cytotoxicity at 10 μg/ml in RAW264.7 cells, and therefore subsequent studies were carried out at 10 μg/ml silica particle treatment.

**Figure 2:**
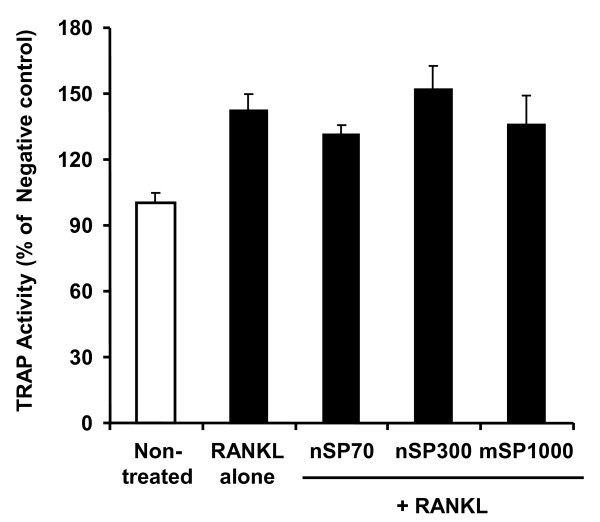
**Effect of silica particles on the RANKL-induced TRAP activity in RAW264.7 cells**. RAW264.7 cells were incubated with 10 μg/ml silica particles and 30 ng/ml RANKL for 5 days. TRAP activity was calculated relative to the negative controls (medium without RANKL). Data are expressed as the mean ± SD (*n *= 3).

**Figure 3:**
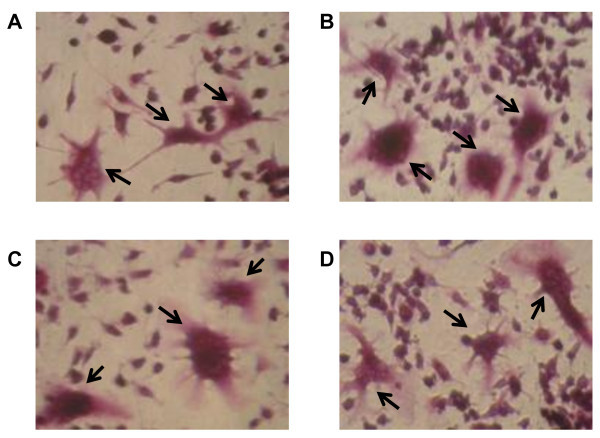
**Effect of silica particles on the RANKL-induced osteoclast differentiation in RAW264.7 cells (TRAP staining)**. RAW264.7 cells were incubated with 10 μg/ml silica particles and 30 ng/ml RANKL for 5 days. Osteoclast genesis was confirmed by TRAP staining. (**A**) RANKL alone (30 ng/ml), (**B**) nSP70 10 μg/ml with RANKL 30 ng/ml, (**C**) nSP300 10 μg/ml with RANKL 30 ng/ml, (**D**) mSP1000 10 μg/ml with RANKL 30 ng/ml. Arrows show osteoclast cells (TRAP-positive multinucleate cells (> 3 nuclei)). Magnifications of all photographs are ×400.

**Figure 4:**
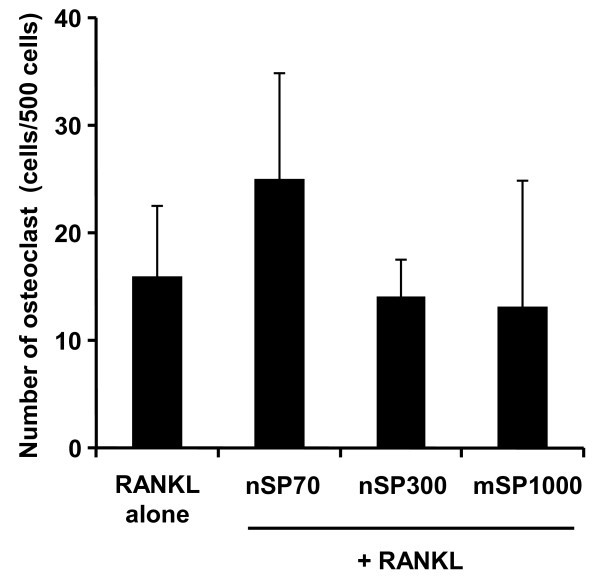
**Number of osteoclasts induced by RANKL and silica particles in RAW264.7**. RAW264.7 cells were incubated with 10 μg/ml silica particles and 30 ng/ml RANKL for 5 days. The number of TRAP-positive multinucleate cells (> 3 nuclei) were counted as osteoclasts in three different areas. Data are expressed as the mean ± SD (*n *= 3).

In order to investigate the effects of nanosilica on osteoclast differentiation in RAW264.7 cells, TRAP activity, which is a known indicator of osteoclast differentiation, was measured. TRAP activity levels of RANKL (which is a key molecule in osteoclast differentiation)-treated RAW264.7 cells were significantly increased compared to non-treated RAW264.7 cells (Figure 2). On the other hand, TRAP activity levels of RAW264.7 cells treated with RANKL and silica particles were almost equal to those of cells treated with RANKL alone. An analysis comparing TRAP activities between nSP70, nSP300 and mSP1000 did not reveal significant size-dependent changes (Figure 2). Furthermore, the number of TRAP-positive multinucleated cells were tallied as a measure of osteoclast differentiation by RANKL and silica particle treatment. As a result of TRAP staining, RAW264.7 cells treated by RANKL alone or by RANKL with silica particles were almost all TRAP positive (Figure 3). The number of TRAP-positive multinucleated cells indicated no significant differences following each silica particle treatment, although nSP70-treated RAW264.7 cells tended to have an increased number of TRAP-positive multinucleated cells compared to cells treated with RANKL alone (Figure 4). These results suggested that none of the silica particle sizes used in this study induced effects on osteoclast differentiation of RAW264.7 cells at the doses given.

In the dentistry field, it is well-known that wear particles from materials used in implants can induce bone destruction, and this can become a significant therapeutic problem in the treatments requiring implants and/or artificial bone. Some reports have suggested that micro-sized particles (about 4-9 μm in diameter) increased the expression of RANKL and/or osteoclast differentiation-related genes in bone marrow cells [16,17]. These reports suggested that micro-sized particles above 4 μm accelerated the osteoclast differentiation. On the other hand, in the case of nano- and subnano-sized particles, it has been reported that particles did not affect osteoclast differentiation or the expression of inflammatory cytokines and osteoclast differentiation-regulated genes [18]. Our earlier results and the present report suggest that acceleration effects of particles on osteoclast differentiation slowed as the particles got smaller, until they reached nanosize. In other words, the particle size is an important characteristic in the ability of nanoparticles to induce osteoclast differentiation. The mechanism by which cells take up particles differs according to particle sizes [19]. The larger particles (from several micrometers to several dozen micrometers) and the smaller particles (from several dozen to several hundred nanometers) are believed to be taken up by macrophage cells via phagocytosis and macropinocytosis, respectively [19]. Therefore, these differences of particle uptake pathway may be one of the factors producing the observed effect of particle size on osteoclast differentiation.

Collectively, the results presented here indicated that nanosilica has no effect on osteoclast differentiation. They also support the safety of nanosilicas that are used in the production of highly functional materials for oral care fields [1,20]. We believe that applications of nanosilica will extend to these new fields in the near future following further careful safety study.

## Competing interests

The authors declare that they have no competing interests.

## Authors' contributions

HN and TY designed the study. HN, TA, TY, ST, TH, MU, KI, TY, KH and YM performed experiments. HN and TY collected and analysed data. HN and TY wrote the manuscript. KN, YA, HK, ST, NI and YY gave technical support and conceptual advice. YT supervised all of the projects. All authors discussed the results and commented on the manuscript.
